# Integrated Network Analysis of microRNAs, mRNAs, and Proteins Reveals the Regulatory Interaction between hsa-mir-200b and CFL2 Associated with Advanced Stage and Poor Prognosis in Patients with Intestinal Gastric Cancer

**DOI:** 10.3390/cancers15225374

**Published:** 2023-11-12

**Authors:** Everton Cruz dos Santos, Paulo Rohan, Renata Binato, Eliana Abdelhay

**Affiliations:** Stem Cell Laboratory, Division of Specialized Laboratories, Instituto Nacional de Câncer (INCA), Rio de Janeiro 20230-130, RJ, Brazil; rohanphn@gmail.com (P.R.); renata.binato@inca.gov.br (R.B.); eabdelhay@inca.gov.br (E.A.)

**Keywords:** intestinal gastric cancer, bioinformatics, transcriptomics, proteomics, miRNomics, regulatory network

## Abstract

**Simple Summary:**

To understand the bionetworks involved in intestinal gastric cancer (IGC), an integrative approach to investigate omics data is essential; otherwise, a broad comprehension of IGC molecular networks cannot be achieved. Thus, an integrative analysis of high-throughput data from the expression of major biological molecules (mRNAs, microRNAs, and proteins) was performed to identify key components in the development of IGC, which had not been performed until now. Through the identification of regulatory circuits based on the interaction of microRNA/mRNA/proteins, we were able to identify different mechanisms of gene regulation impacting similar biological functions and key regulators (hubs) in IGC development. Besides their good potential as diagnostic, prognostic, and tumor development biomarkers, hub expression was related to relevant aspects of IGC patient clinicopathology, where we found hub interactions operating together to impact clinical outcomes. This approach will help us better understand the biological and clinical relevance of complex molecular bionetwork in IGC.

**Abstract:**

Intestinal gastric cancer (IGC) carcinogenesis results from a complex interplay between environmental and molecular factors, ultimately contributing to disease development. We used integrative bioinformatic analysis to investigate IGC high-throughput molecular data to uncover interactions among differentially expressed genes, microRNAs, and proteins and their roles in IGC. An integrated network was generated based on experimentally validated microRNA-gene/protein interaction data, with three regulatory circuits involved in a complex network contributing to IGC progression. Key regulators were determined, including 23 microRNA and 15 gene/protein hubs. The regulatory circuit networks were associated with hallmarks of cancer, e.g., cell death, apoptosis and the cell cycle, the immune response, and epithelial-to-mesenchymal transition, indicating that different mechanisms of gene regulation impact similar biological functions. Altered expression of hubs was related to the clinicopathological characteristics of IGC patients and showed good performance in discriminating tumors from adjacent nontumor tissues and in relation to T stage and overall survival (OS). Interestingly, expression of upregulated hub hsa-mir-200b and its downregulated target hub gene/protein CFL2 were related not only to pathological T staging and OS but also to changes during IGC carcinogenesis. Our study suggests that regulation of CFL2 by hsa-miR-200b is a dynamic process during tumor progression and that this control plays essential roles in IGC development. Overall, the results indicate that this regulatory interaction is an important component in IGC pathogenesis. Also, we identified a novel molecular interplay between microRNAs, proteins, and genes associated with IGC in a complex biological network and the hubs closely related to IGC carcinogenesis as potential biomarkers.

## 1. Introduction

Gastric cancer (GC) has the fifth highest incidence and fourth highest mortality globally, with 1089.103 new cases and 768,793 related deaths in 2020 [1]. Late detection, which is mainly due to nonspecific symptoms in early stages, is one of the main problems associated with GC and is directly related to poor prognosis and the short five-year survival rate of approximately 20% of cases. Furthermore, surgical resection and chemotherapy have limited value in advanced-stage patients [2,3,4,5]. Lauren’s classification [6] divides adenocarcinoma into two subtypes, diffuse and intestinal, which differ in their clinical and epidemiological features. Intestinal gastric cancer (IGC) can develop over years and originate from the evolution of sequential lesions; it is also the most prevalent subtype worldwide. To understand the molecular alterations involved in GC, omics studies such as metabolomics, transcriptomics, and proteomics have been performed; however, most of these studies did not employ more than one technique, which limits a broad understanding of how molecular background and functional networks are related to IGC.

In recent years, important studies integrating data from expression of mRNAs and microRNAs to identify microRNA targets have been performed in many cancer models [7,8,9,10] and in gastric cancer, e.g., prediction of interactions between mRNAs and noncoding RNAs [11,12,13,14]. Although this approach is very important for understanding gene-microRNA relationships, it is not sufficient to understand the global process of gene expression as all these processes are controlled by a complex relationship between miRNAs, mRNAs, and proteins. In 2014, The Cancer Genome Atlas (TCGA) program conducted a large study using high-throughput molecular approaches such as whole-exome sequencing and mRNA sequencing and was able to identify molecular subtypes of GC [15]. These data are accessible and can be utilized in integrative studies. However, there are scarce protein data in the TCGA database. In fact, only a few studies have integrated data from high-throughput analyses of differentially expressed genes (DEGs), microRNAs (DEMs), and proteins (DEPs) [16,17,18], and such studies in cancer models are rare [19]. Moreover, no studies on IGC have integrated these data.

Because high-throughput studies often generate a large volume of data, bioinformatics approaches combined with expression profile analyses can be a crucial tool for understanding the relationship between those molecules and their role in IGC, particularly pathways in the interaction network. In this study, high-throughput approaches (proteomic, miRNomics, and transcriptomic) and integrative bioinformatic analysis were combined to explore and investigate the altered expression profiles of DEGs, DEMs, and DEPs in tumor tissues compared with adjacent nontumor tissues of IGC patients. We used microRNA and mRNA data collected from TCGA and integrated them with data from our previous proteomic study to uncover the main networks related to IGC. We identified three regulatory circuits involved in a complex interaction network that contribute to progression of IGC. Key regulators were determined from those networks, including 23 DEM and 15 DEG/DEP hubs. The regulatory circuit networks were associated with the hallmarks of cancer, such as cell death, apoptosis, the cell cycle, the immune response, and epithelial-to-mesenchymal transition, through potentially different regulatory mechanisms. Additionally, pathways related to KRAS, PI3K/AKT/mTOr, and p53 signaling were found to be enriched. Altered expression of some identified hubs correlated with the clinicopathological characteristics of IGC patients and showed good performance in discriminating tumors from adjacent nontumor tissues. Furthermore, the levels of three DEM hubs, hsa-miR-17, hsa-miR-373, and hsa-miR200b, and two DEG hubs, *CFL2* and *QSOX2*, were significantly associated with overall survival in IGC patients. Interestingly, upregulated expression of hsa-mir-200b and downregulated expression of its target *CFL2* in one of the circuits were found to be related not only to pathological T staging but also to patient overall survival, indicating that these may be important components in the pathogenesis of IGC. The findings relating to this integrative network will help us better understand the complex molecular bionetwork of IGC, highlighting new potential targets for further investigation.

## 2. Materials and Methods

### 2.1. Acquisition of Gastric Cancer Datasets, Patient Samples, and Study Design

**Figure 1:**
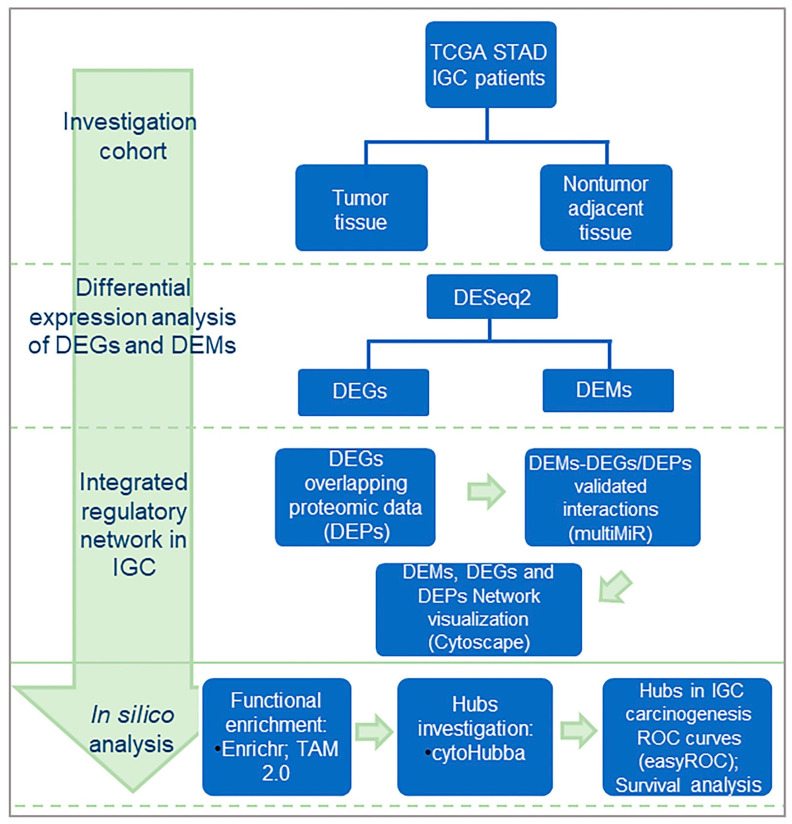
Study design. Data from tumor tissues and adjacent nontumor tissue controls from IGC were obtained from the TCGA database. Differential expression analysis of DEMs and DEGs was performed. The DEGs were compared with the DEPs from our previous study. Common identifications were used as guides for construction of a network with integration of validated DEM interaction data, resulting in the construction of three networks regarded as regulatory circuits. The list of identifications was subjected to functional enrichment analyses, and the hubs for each circuit were identified. Finally, the impact of hub expression on IGC carcinogenesis was evaluated. IGC = intestinal gastric cancer; DEMs = differentially expressed microRNAs; DEGs = differentially expressed genes; DEPs = differentially expressed proteins.

In this study, RNA-seq and clinical data for tumor tissues (*n* = 180) and adjacent nontumor tissues (*n* = 18) from IGC patients were obtained from The Cancer Genome Atlas (TCGA) project [15]. Additionally, data for DEPs were obtained from our previous work [20] from patients diagnosed with IGC (Supplementary Table S1). The included samples were investigated according to the design shown in Figure 1. The TCGA IGC patient characteristics are summarized in Table 1.

### 2.2. Differential Expression Analysis of DEGs and DEMs

The clinical data and expression quantification of miRNAs and mRNAs for 180 tumor tissue samples and 18 adjacent nontumor tissue samples from IGC patients publicly available in TCGA (TCGA-STAD) were downloaded through the R software (version 3.6.1, https://www.r-project.org/, accessed on 5 March 2023) (R software, RRID:SCR_001905) using the package TCGAbiolinks (TCGAbiolinks, RRID:SCR_017683) [21] from R/Bioconductor (http://www.bioconductor.org/, accessed on 5 March 2023) (Bioconductor, RRID:SCR_006442). Sample normalization and DEG and DEM identification were performed using the DESeq2 (DESeq2, RRID:SCR_015687) [22] package. An adjusted *p* value ≤ 0.05 and |log2FC| ≥ 1 were considered indicative of differential expression for DEG selection, and an adjusted *p* value ≤ 0.05 and |log2FC| > 0 were applied to select DEMs.

### 2.3. Prediction of microRNA and mRNA Interactions

To obtain DEM–DEG pairs, validated interactions between DEMs and target DEGs were acquired using the multiMiR [23] package, including 3 databases (miRecords, miRTarBase, and TarBase) with available validated interactions. Genes with interactions that have been validated and not annotated as “negative” in the “support_type” field by miRTarBase were considered miRNA targets. DEMs and DEGs from these analyses were selected for downstream investigations.

### 2.4. Proteomic Data for IGC Patients

Differentially expressed protein data were obtained from a previous work by our group [20]. Total protein was extracted from tumor and adjacent nontumor tissues and analyzed using a label-free nano LC–MS/MS approach. The samples were subjected to nanoscale chromatographic separation (2DnanoLC) using the nanoACQUITY UPLC system from Waters^®^, followed by mass spectrometry using a Synapt HDMS mass spectrometer (Waters, Wilmslow, UK). A total of 429 DEPs were identified [20].

### 2.5. In Silico Analysis and Integration Network of Differentially Expressed microRNAs, Genes, and Proteins

The DEM–DEG–DEP regulatory network was constructed and visualized using Cytoscape (version 3.9.1) (Cytoscape, RRID:SCR_003032) [24]. Functional enrichment analysis of DEGs/DEPs was performed using Enrichr (RRID:SCR_001575) [25], and miRNA enrichment was performed using TAM 2.0: tool for microRNA set analysis [26,27]. These tools apply overrepresentation analysis (ORA), and the *p* value obtained was corrected using FDR from Benjamini-Hochberg adjustment [28] for each category independently, with significant categories being identified for adjusted *p* values ≤ 0.05.

IGC hubs were determined through the Cytoscape plugin “cytoHubba” (cytoHubba, RRID:SCR_017677) using the maximal clique centrality (MCC) method for DEGs/DEPs [29]. The tool “Analyze network” from Cytoscape was used for DEMs. This tool allows analysis of “direct networks”, extracting the number of direct interactions of regulators and targets, i.e., the out-degree for the regulators and the in-degree for the targets [30]. DEMs with the highest number of out-degrees were considered hubs; in the event of an equal number, all hubs with the same number of targets were selected. The relationship between the identified hubs and patient clinicopathological characteristics was tested using the Kruskal-Wallis test; pairwise comparisons were tested using the Mann-Whitney test. Additionally, correlation between expression levels of selected DEG–DEM hub pairs was assessed using Pearson’s correlation.

### 2.6. ROC Curves and Survival Analysis of DEM and DEP Hubs

The potential diagnostic power of DEM and DEG hubs was determined by receiver operating characteristic (ROC) curve analysis using the easyROC software (ver. 1.3.1) [31] with default parameters. The area under the ROC curve (AUC) was computed to measure the performance of each DEM and DEG individually in differentiating tumors from normal samples. The AUC is a measurement of the overall performance of a diagnostic test and has a value between 0 and 1. If the area under the ROC curve is 1, the test operates with 100% sensitivity (no false-negative results) and 100% specificity (no false-positive results). An AUC closer to 1 corresponds to better test performance, whereas a value of 0.5 indicates that the model performance is no better than random chance. AUC values of 90–100 = excellent, 80–90 = good, 70–80 = fair, 60–70 = poor, and 50–60 = failure [32,33]. For survival analysis, patients were divided into low- and high-expression groups based on the values of gene expression that were processed with the method of variance stabilizing transformation [22]. Based on this value, the low-expression group was established as the lower quartile of expression and the high-expression group as the upper quartile of expression, with 45 IGC patients per group. Overall survival analysis was performed using the survival package (version 2.11-4) (survival, RRID:SCR_021137) through the Kaplan-Meier method. Log-rank test *p* values ≤ 0.05 were considered statistically significant.

## 3. Results

### 3.1. Identification of DEGs and DEMs in IGC Patients

**Figure 2:**
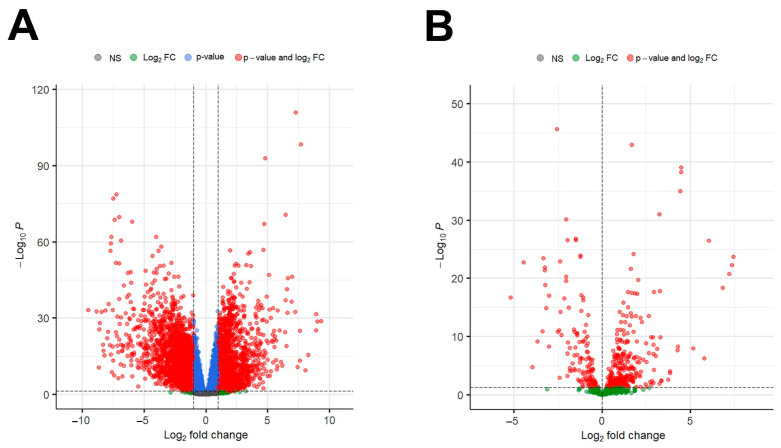
Differential gene expression analysis of genes and miRNAs of IGC patients from the TCGA-STAD dataset. (**A**) Volcano plot of mRNAs in IGC patients. (**B**) Volcano plot of microRNAs in IGC patients. The *y*-axis represents the negative base 10 logarithm of the adjusted *p* value, and the *x*-axis represents the log2−fold change. An adjusted *p* value ≤ 0.05 and |log2FC| ≥ 1 were considered indicative of differential expression for DEG selection; an adjusted *p* value ≤ 0.05 and |log2FC| > 0 were applied to select DEMs. The red dots correspond to individual DEGs (**A**) and DEMs (**B**) with significant expression differences (combining *p* value and log2FC cutoff) (24.73% for DEGs and 22.7% for DEMs). The green dots correspond to mRNAs or microRNAs that reached the minimum value of log2FC but not the *p* value (1.55% for DEGs and 77.3% for DEMs). The blue dots correspond to mRNAs or microRNAs that reached the minimum *p* value but not log2FC (36.64% for DEGs and none for DEMs). The gray dots correspond to nonsignificant mRNAs or microRNAs (37.07% for DEGs and none for DEMs). IGC = intestinal gastric cancer; DEMs = differentially expressed microRNAs; DEGs = differentially expressed genes; DEPs = differentially expressed proteins.

For this study, samples from 180 IGC tissues and 18 adjacent normal tissues were selected from the TCGA database (Table 1), from which DEGs and DEMs were obtained. A total of 4902 DEGs, including 2220 upregulated (*p* value ≤ 0.05, log2FC ≥ 1) and 2682 downregulated (*p* value ≤ 0.05, log2-fold change ≤ −1) DEGs (Figure 2A), were identified. Additionally, 427 DEMs (*p* value ≤ 0.05, |log2FC| > 0) were identified. In contrast to the DEGs, more DEMs were upregulated than downregulated (297 and 130, respectively) (Figure 2B). The lists of all selected DEMs and DEGs are provided in Supplementary Tables S2 and S3.

### 3.2. Construction of an Integrated Regulatory Network in IGC

#### 3.2.1. DEG and DEP Selection

To verify possible regulatory mechanisms related to molecular alterations in IGC, we constructed interaction networks between expression of microRNAs, mRNAs, and proteins that might explain the regulatory background related to the disease. To construct the integrated regulatory networks of DEMs, DEGs, and DEPs, we first compared the lists of 4902 DEGs and the 429 DEPs from our previous proteomic study. We chose this specific approach because if DEPs are indeed the result of gene expression related to the disease, they are a good guiding characteristic to build a network. We only selected common identification between DEGs and DEPs that we regarded as markers of the disease once they were identified both in the TCGA cohort (at the mRNA level) and in our cohort (at the protein level), thus validating expression of those genes. Additionally, by using our protein data, we overcame the limited protein information available in TCGA.

**Figure 3:**
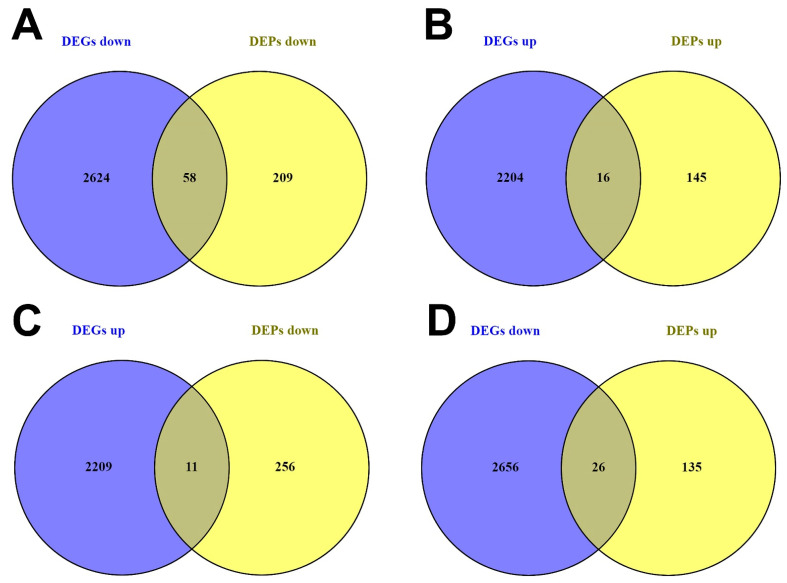
Overlap identification between DEGs and DEPs. Common findings (DEGs/DEPs) were segregated into four main groups. (**A**) Comparison between downregulated DEGs and downregulated DEPs. (**B**) Comparison between upregulated DEGs and upregulated DEPs. (**C**) Comparison between upregulated DEGs and downregulated DEPs. (**D**) Comparison between downregulated DEGs and upregulated DEPs. DEGs = differentially expressed genes; DEPs = differentially expressed proteins.

After overlap comparison between the identified DEGs and DEPs, common findings were segregated into four main groups. The first group was obtained through a comparison between downregulated DEGs (2682) and downregulated DEPs (267), which resulted in 58 common downregulated “DEGs/DEPs” characterized by downregulation at both the mRNA and protein levels (Figure 3A). The second group was characterized by comparison between upregulated DEGs (2220) and upregulated DEPs (161), resulting in 16 common DEGs/DEPs (Figure 3B). The third group was obtained by comparison between upregulated DEGs and downregulated DEPs, leading to identification of 11 DEGs/DEPs with divergent expression between mRNA and proteins (Figure 3C). The last group was based on comparison of downregulated DEGs and upregulated DEPs, which resulted in 26 DEGs/DEPs, also with divergent expression between mRNA and proteins (Figure 3D. We performed the next steps involved in the regulatory network construction based on these preselected groups.

#### 3.2.2. Distinctly Altered Regulatory Circuits Involving DEMs, DEGs, and DEPs Are Related to IGC

To investigate important specific regulatory interactions that might explain the observed expression levels of DEGs and DEPs, we also included information about DEM expression in the network. This may constitute a regulatory link connecting the observed altered expression of DEGs, resulting in an alteration in DEPs. Nevertheless, to include DEMs, we decided to continue the investigation with the interaction pairs that followed an important biological attribute, which is the classical regulatory mechanism of microRNAs. This mechanism depends on the complementarity of miRNA and target mRNA: when complementarity with the target mRNA is perfect, the target is degraded by the miRNA–RISC machinery, resulting in mRNA downregulation; when complementarity is partial, it promotes translation repression, resulting in decreases in the protein level without changing the mRNA level [34]. This fundamental regulatory mechanism was used as a rationale for creating the networks.

In this way, the DEMs targeting the DEGs with protein-level expression that were verified in our proteomic work were selected and included in the interaction as a regulatory component, potentially explaining the deregulated status of the DEGs and DEPs. To give more strength to our study, we included in the network only the interaction pairs of DEMs–DEGs with available experimentally validated interactions as investigated in three databases (miRecords, miRTarBase, and TarBase). Thus, the final networks were built based on the overlapping DEGs and DEPs targeted by validated regulatory DEMs, thereby accounting for the classical mechanism of regulation through microRNAs.

**Figure 4:**
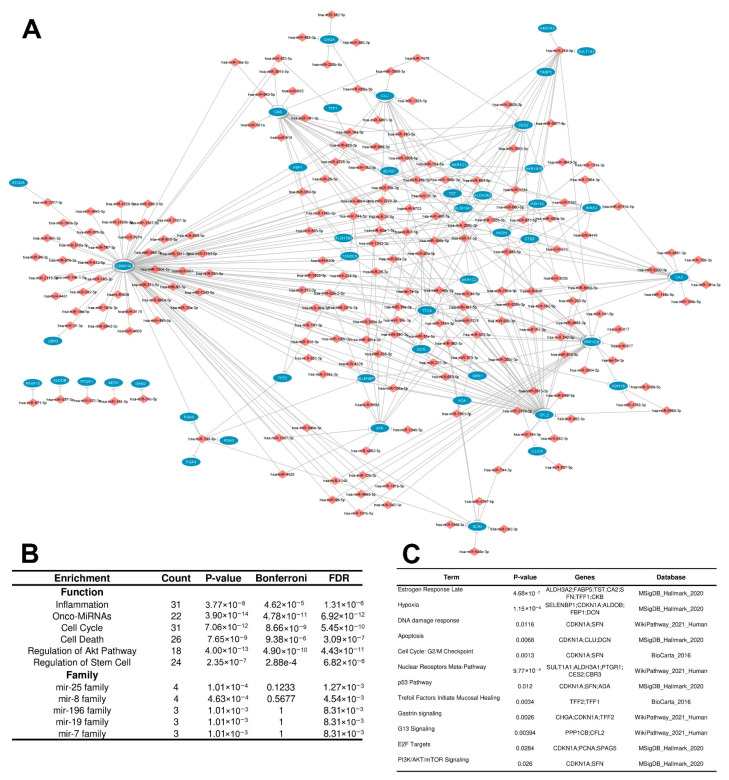
Interaction network and enrichment analysis regarding the first repressive regulatory circuit (RRC1) of DEMs and DEGs/DEPs in IGC. (**A**) RRC1 is defined by a network involving 399 interactions between upregulated DEMs (199) targeting downregulated DEGs/DEPs (48). Ellipses represent DEGs/DEPs, and diamond forms represent DEMs. Expression node levels are presented by the colors red (increased expression) and blue (decreased expression). Gray lines indicate inhibition. (**B**) DEM enrichment analysis performed using the TAM 2.0 software. (**C**) DEG/DEP enrichment analysis performed using the Enrichr tool software version 3.2. IGC = intestinal gastric cancer; DEMs = differentially expressed microRNAs; DEGs = differentially expressed genes; DEPs = differentially expressed proteins.

**Figure 5:**
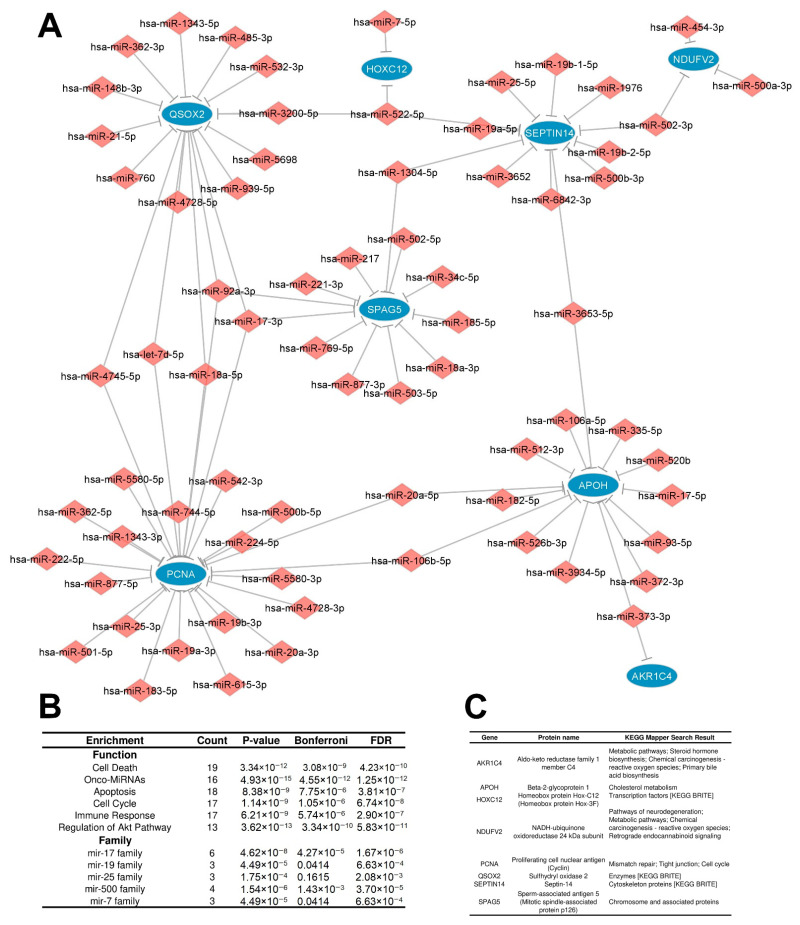
Interaction network and enrichment analysis regarding the second repressive regulatory circuit (RRC2) of DEMs and DEGs/DEPs in IGC. (**A**) RRC2 is defined by a network involving 86 interactions between upregulated DEMs targeting upregulated DEGs with downregulated DEPs. Ellipses represent DEGs/DEPs, and diamond forms represent DEMs. Expression node levels are presented by the colors red (increased expression) and blue (decreased expression only shown regarding DEPs). Gray lines indicate inhibition. (**B**) DEM enrichment analysis performed using the TAM 2.0 software. (**C**). DEG/DEP enrichment analysis was performed using the KEGG database. IGC = intestinal gastric cancer; DEMs = differentially expressed microRNAs; DEGs = differentially expressed genes; DEPs = differentially expressed proteins.

**Figure 6:**
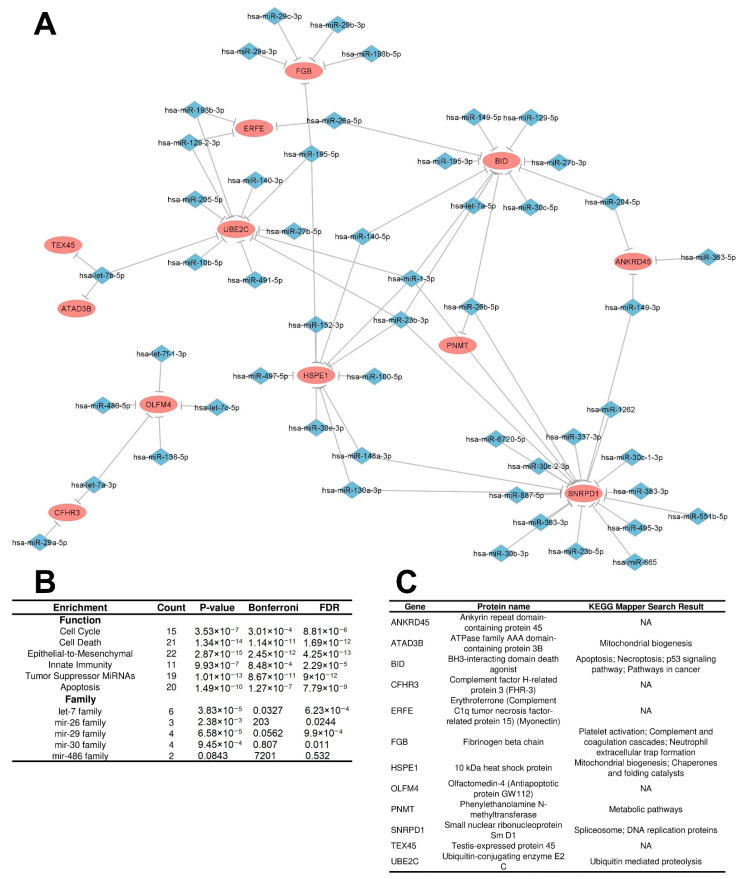
Interaction network and enrichment analysis regarding the third inductive regulatory circuit (IRC) of DEMs, DEGs, and DEPs in IGC. (**A**). IRC is defined by a network involving 73 interactions between downregulated DEMs targeting upregulated DEGs/DEPs. Ellipses represent DEGs/DEPs, and diamond forms represent DEMs. Expression node levels are presented by the colors red (increased expression) and blue (decreased expression). Gray lines indicate inhibition. (**B**) DEM enrichment analysis performed using the TAM 2.0 software. (**C**). DEG/DEP enrichment analysis was performed using the KEGG database. IGC = intestinal gastric cancer; DEMs = differentially expressed microRNAs; DEGs = differentially expressed genes; DEPs = differentially expressed proteins.

Three networks met these criteria, characterizing three potential regulatory circuits: two repressive and one inductive. The first repressive regulatory circuit (RRC1) was defined by a network involving 399 interactions between upregulated DEMs (199) targeting downregulated DEGs/DEPs (48), which are potentially related to direct degradation of DEGs by DEMs, leading to the downregulation observed at both the mRNA and protein levels (Figure 4A). The second repressive regulatory circuit (RRC2) was characterized by a network involving 86 interactions between upregulated DEMs (71) targeting upregulated DEGs (8) with downregulated DEPs (8), which we presume are potentially related to translation repression of DEGs by DEMs, leading to downregulation being observed only at the protein level (Figure 5A). The third inductive regulatory circuit (IRC) was characterized by a network involving 73 interactions between 54 downregulated DEMs targeting upregulated DEGs/DEPs (12) (Figure 6A), leading to upregulation of both the mRNA and protein levels because their DEM regulator was repressed. To avoid confounding factors, we did not create a fourth circuit with the microRNAs related to downregulated DEGs and upregulated proteins (26); such interactions are not in accordance with the rationale used for the previous circuits.

### 3.3. Functional Enrichment Analysis of Regulatory Circuits

The identified regulatory circuits showed a very specific composition in terms of DEM and DEG/DEP interactions and are potentially related to specific functions that may characterize and promote carcinogenesis in IGC patients. To investigate their functional roles in the circuit, we performed enrichment analysis using the TAM 2.0 software, which provided us with an overview of the important functions in which the DEMs are enriched. Enrichment analysis for DEGs/DEPs was performed with the Enrichr tool, which enables enrichment in several databases simultaneously.

The RRC1 enrichment analysis (characterized by upregulated DEMs targeting downregulated DEGs/DEPs) showed that the DEMs with induced expression in tumors were enriched mainly in the onco-MiRNA class, followed by regulation of the Akt pathway, the cell cycle, cell death, inflammation, and regulation of stem cell, indicating its protumorigenic regulation activities. Additionally, specific microRNA families were enriched. These families are important because they might control specific pathways and share similar functions. The mir-25 family and mir-8 family were the most enriched families, followed by the mir-196, mir-19, and mir-7 families (Figure 4B). In turn, the target downregulated DEMs/DEPs in RRC1 were enriched in cell metabolism processes, such as the cell cycle, apoptosis, and functioning of the stomach, as well as important pathways related to cancer, such as KRAS, PI3K/AKT/mTOr, and p53 signaling (Figure 4C).

The enrichment analysis for RRC2 (characterized by interactions between upregulated DEMs targeting upregulated DEGs with downregulated DEPs) showed that the DEMs were related to the same biological function as the first regulatory circuit (onco-miRNAs, regulation of the Akt pathway, cell death, the cell cycle, the immune response, and apoptosis) (Figure 5B). This may be because 73% (52/70) of the set of microRNAs in both circuits were equal; it may also indicate that the same DEMs potentially regulate different targets through different mechanisms, that is, through direct degradation of DEMs in RRC1 and through inhibition of translation in RRC2. This might explain why the DEGs were upregulated while the corresponding DEPs were downregulated in RRC2. Nevertheless, the most enriched microRNA families differed. mir-17 and mir-500 were the top families, followed by the mir-19, mir-7, and mir-25 families, indicating that different DEM families regulate different circuits. The number of DEGs/DEPs in RRC2 was small, limiting the statistical power to perform a proper enrichment analysis. Thus, we chose to verify the pathways to which these players map in the KEGG database. The results indicated that the proteins mapped to important processes, such as chemical carcinogenesis, mismatch repair, the cell cycle, and other important metabolic processes (Figure 5C).

Enrichment analysis of the IRC components (characterized by downregulated DEMs targeting upregulated DEGs/DEPs) showed interesting results. First, we observed that the epithelial-to-mesenchymal transition and tumor-suppressor miRNAs were enriched with the smallest *p* values and FDR. Second, a repetition of enriched biological functions was shown for the upregulated DEMs in the two repressive regulatory circuits: cell death, apoptosis, and the cell cycle (Figure 6B). These results emphasize the following: the downregulated DEMs fail to repress the activities of tumor-promoter proteins that are potentially involved in the same process regulated by the upregulated DEMs in the repressive regulatory circuits. This indicates that there are complex regulatory dynamics involving tumor-suppressor miRNAs and onco-miRNAs regulating the same biological functions that seem to be central in IGC. Furthermore, the most prominent families enriched were the let-7, mir-29, and mir-30 families. As observed for RRC2, the number of DEGs/DEPs was also small. Nevertheless, we observed proteins mapping to important processes, such as apoptosis, necroptosis, DNA replication, immune activities, and protein metabolism (Figure 6C).

### 3.4. Identification of Key Regulators

Integrated networks from high-throughput data generate much information, which complicates interpretation of the data. To reduce the complexity of information and elucidate the main regulatory components of our networks, finding key components through identification of hubs is a useful approach.

As hubs are characterized by many interactions, a given DEM hub can regulate several DEGs/DEPs, and a DEG hub is targeted by several DEMs. Additionally, they commonly display important roles in multiple pathways given their large number of interactions; thus, they are important key regulators of disease development. To identify the hubs in our data, the highly connected molecules for each regulatory circuit were determined. The plug-in “cytoHubba” in Cytoscape was used to rank the nodes by their network features through topological analysis methods. The DEG/DEP hubs were chosen based on the top 5 nodes from the network identified by the maximal clique centrality (MCC) analysis method, which has good performance regarding prediction precision [29]. The results revealed 15 DEG/DEP hubs: five in RRC1 (CDKN1A, PPP1CB, CKB, TTC9, and CFL2), five in RRC2 (PCNA, QSOX2, APOH, SPAG5, and SEPTIN14) and five in IRC (SNRPD1, BID, UBE2C, HSPE1, and OLFM4) (Table 2). For DEM hub identification, we used the tool “Analyze network” in the Cytoscape software, that allows for analysis of “direct networks”, which describe regulatory networks.

DEM hubs were identified by the “out-degree” (number of targets); importantly, when an equal number was obtained, all hubs with the same number of targets were selected. The results showed 10 hubs in the first repressive circuit (hsa-miR-210-3p, hsa-miR-200b-3p, hsa-miR-335-5p, hsa-miR-20a-5p, hsa-miR-148b-3p, hsa-miR-17-5p, hsa-miR-15b-5p, hsa-miR-15a-5p, hsa-miR-106b-5p, and hsa-miR-92a-3p), 12 hubs in the second repressive circuit (hsa-miR-17-3p, hsa-miR-522-5p, hsa-miR-92a-3p, hsa-let-7d-5p, hsa-miR-18a-5p, hsa-miR-3653-5p, hsa-miR-373-3p, hsa-miR-4745-5p, hsa-miR-20a-5p, hsa-miR-106b-5p, hsa-miR-1304-5p, and hsa-miR-502-3p) and 5 hubs in the third inductive circuit (hsa-miR-23b-3p, hsa-miR-1-3p, hsa-let-7b-5p, has-miR-195-5p, and hsa-miR-26b-5p). Interestingly, in each circuit, some hubs regulated a high proportion of targets, which indicates their importance in the network (Table 3).

### 3.5. Impact of Hubs in IGC Carcinogenesis

Once the hubs in the circuits are identified, it is important to investigate how their deregulated expression in IGC might be related to several clinicopathological aspects that are important to patients as well as to knowledge of disease. Thus, we evaluated the association of hubs with diagnostic power, clinicopathological characteristics, and prognostic prediction.

#### 3.5.1. Diagnostic Value of Key Hubs in IGC

**Figure 7:**
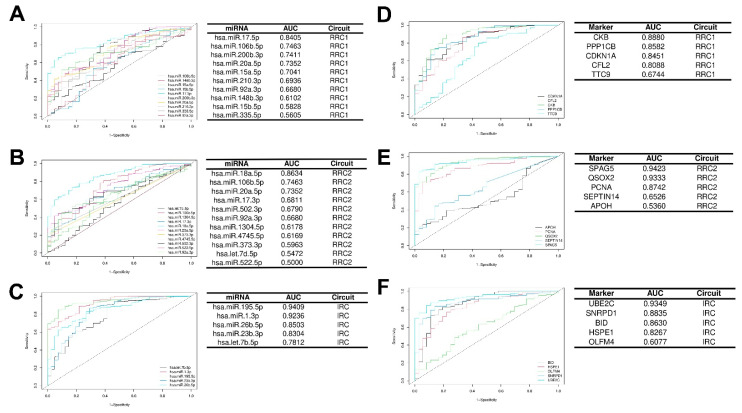
ROC curve analysis of hubs. ROC curves were constructed using the easyROC software based on expression of hubs in tumor vs. adjacent nontumor tissue in TCGA-STAD IGC patients. ROC curves for DEM hubs of RRC1 (**A**), RRC2 (**B**), and IRC (**C**). ROC curves for DEG hubs of RRC1 (**D**), RRC2 (**E**), and IRC (**F**). IGC = intestinal gastric cancer; DEMs = differentially expressed microRNAs; DEGs = differentially expressed genes.

ROC curve analysis was performed to determine the discriminative power of deregulated expression of hubs for diagnostic performance. From all DEM hubs, 11 (hsa.miR.17.5p, hsa.miR.106b.5p, hsa.miR.200b.3p, hsa.miR.20a.5p, hsa.miR.18a.5p, hsa.miR.15a.5p, hsa-miR-23b-3p, hsa-miR-1-3p, hsa-let-7b-5p, hsa-miR-195-5p, and hsa-miR-26b-5p) showed good performance in the diagnostic test (areas under the ROC curves (AUCs) > 0.7). The top five AUCs for DEMs were 0.9409 (hsa.miR.195.5p), 0.9236 (hsa.miR.1.3p), 0.8634 (hsa.miR.18a.5p), 0.8503 (hsa.miR.26b.5p), and 0.84051 (hsa.miR.17.5p) (Figure 7A–C), indicating that these DEMs may be good diagnostic markers. From all DEG hubs, 11 (CKB, PPP1CB, CDKN1A, CFL2, SPAG5, QSOX2, PCNA, UBE2C, SNRPD1, BID, and HSPE1) also showed good performance, and the top five AUCs for DEGs were 0.9423 (SPAG5), 0.9349 (UBE2C), 0.9333 (QSOX2), 0.8880 (CKB), and 0.8742 (PCNA); these DEGs may also be good diagnostic markers (Figure 7D–F).

#### 3.5.2. Hub Expression Levels Are Related to Clinicopathological Characteristics and Prognosis in IGC Patients

To verify the relationship of the hubs found in this work with the clinicopathological characteristics of TCGA IGC patients, we verified the association between expression of DEM and DEG hubs with the clinicopathological characteristics of 180 IGC patients in the TCGA cohort.

**Figure 8:**
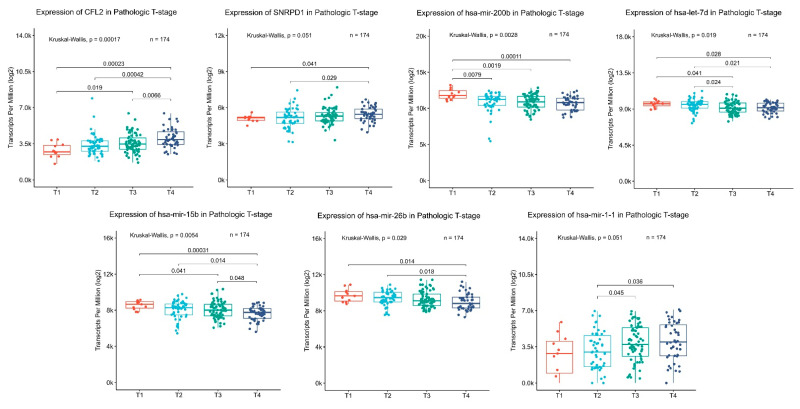
The association of hub expression with different tumor stages from 174 IGC patients in the TCGA cohort. DEMs and DEGs with significant associations are shown: DEMs (hsa-mir-200b, hsa-let-7d, hsa-mir-15b, hsa-mir-26b, and hsa-mir-1-1) and DEGs (*CFL2* and *SNRPD1*). The Kruskal-Wallis test was applied for multiple group comparisons; the Mann-Whitney test was performed for pairwise comparisons. Differences were considered significant at *p* values ≤ 0.05. The pairwise comparison and resulting *p* value between individual stages are represented by horizontal bars. IGC = intestinal gastric cancer; DEMs = differentially expressed microRNAs; DEGs = differentially expressed genes.

**Figure 9:**
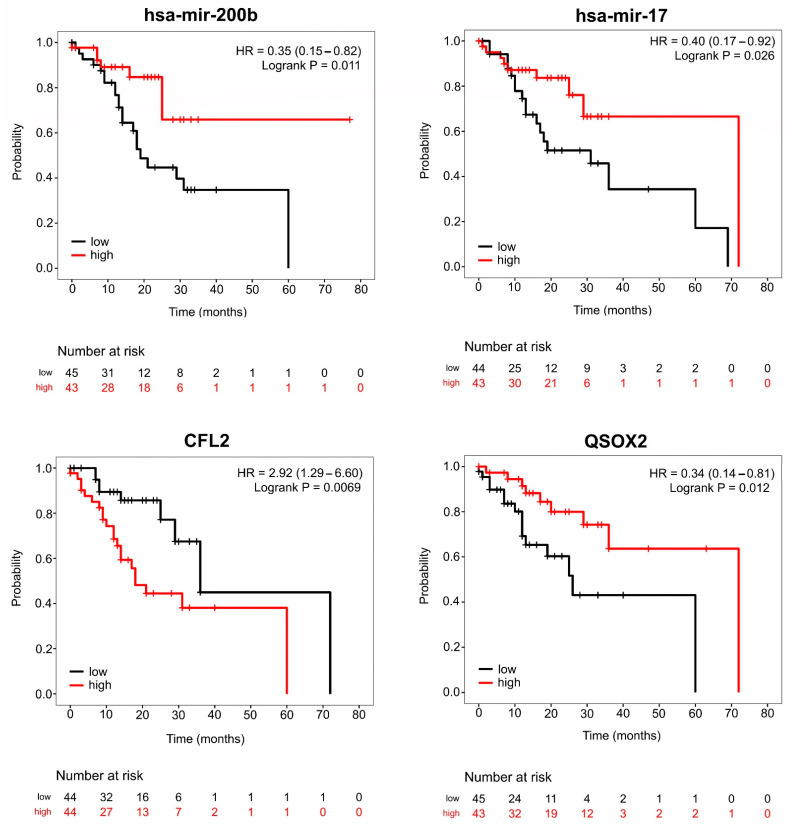
Overall survival of patients stratified according to hub expression. Kaplan-Meier analysis showing the association between hub (hsa-mir-200b, hsa-mir-17, *CFL2*, and *QSOX2*) expression and overall survival (OS) in IGC patients from the TCGA cohort. High- and low-expression groups were divided according to expression quartiles, with the upper quartile representing the high group (red line) and the lower quartile representing the low group (black line). Only those with available survival data were included in the analysis.

Taking the hubs individually, the results showed that expression of two DEGs, upregulated small nuclear ribonucleoprotein Sm D1 (*SNRPD1*) and downregulated cofilin 2 (*CFL2*), was associated with pathological T stage (size of the tumor and any spread of cancer into nearby tissue) (Figure 8). Regarding DEM hubs, we found that altered expression of the upregulated hubs hsa-mir-200b, hsa-mir-15b, and hsa-let-7d and the downregulated hubs hsa-mir-1-1 and hsa-mir-26b was related to pathological T stage (Figure 8). Moreover, we evaluated the prognostic value of the DEM and DEG hubs in the 180 IGC patients of TCGA, and Kaplan–Meier survival curves were used to visualize survival analysis. As depicted in Figure 9, high expression of the DEMs hsa-miR-17 (log-rank *p* value = 0.026) and hsa-miR-200b (log-rank *p* value = 0.011), both from RRC1, were related to better prognosis in IGC patients. Regarding DEGs, we observed that high expression of *CFL2* (log-rank *p* value = 0.0069) (RRC1) and low expression of *QSOX2* (log-rank *p* value = 0.012) (RRC2) were related to poor OS. These data indicate that the identified DEM and DEG hubs are not only the main components in regulatory circuits in IGC carcinogenesis but are also important for patient prognosis and have an impact on overall survival.

### 3.6. Upregulation of hsa-mir-200b Represses Expression of CFL2 and Impacts Prognosis of IGC Patients

**Figure 10:**
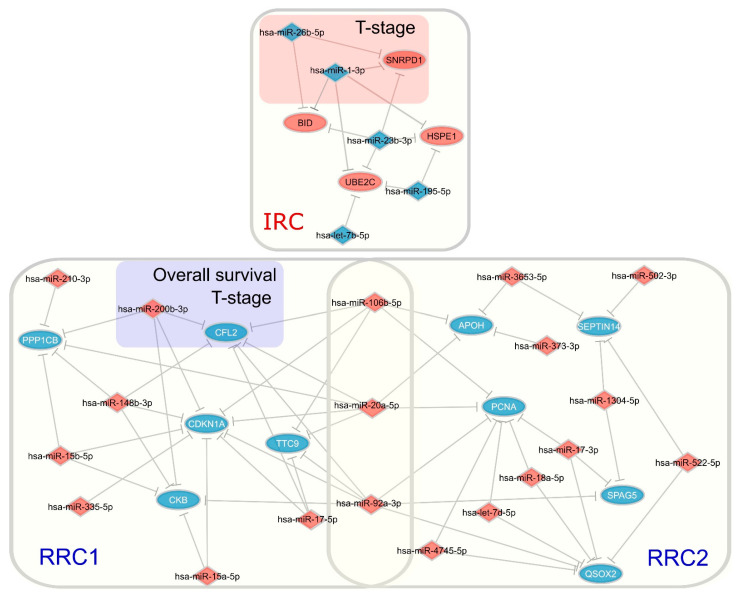
Interactive network of hubs. The regulatory interaction between all 23 DEM and 15 DEG/DEP hubs is shown. Red shaded rectangle in IRC and blue shaded rectangle in IRC1 represent DEM–DEG pairs contributing to clinical outcomes. Ellipses represent DEGs/DEPs, and diamond forms represent DEMs. Expression node levels are presented by the colors red (increased expression) and blue (decreased expression only shown regarding DEPs). Gray lines indicate inhibition. RRC1 = first repressive regulatory circuit; RRC2 = second repressive regulatory circuit IRC = inductive regulatory circuit; DEMs = differentially expressed microRNAs; DEGs = differentially expressed genes; DEPs = differentially expressed proteins.

Interestingly, analyzing the interaction between hubs (Figure 10), we observed that some of the hub components in the regulatory circuits appeared to operate together, contributing to clinical outcomes regarding clinicopathological characteristics. In RRC1, we found that the upregulated DEM hsa-mir-200b hub potentially controlled expression of its target *CFL2*, inducing *CFL2* downregulation at both the mRNA and protein levels, and that this regulatory interaction was significantly related to pathological T stage. The same was observed for IRC, whereby the downregulated DEM hubs hsa-mir-26b and hsa-mir-1-1 failed to inhibit expression of the potential target *SNRPD1,* which was upregulated at both the mRNA and protein levels; they were also significantly related to T stage.

**Figure 11:**
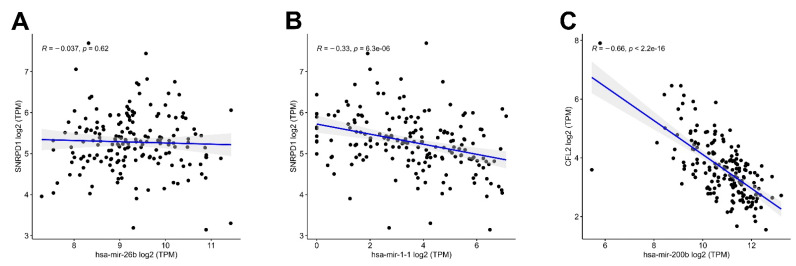
Scatter plots representing correlations among DEM and target DEG hubs in 180 IGC patients of the TCGA cohort. (**A**) hsa-mir-26b and *SNRPD1*. (**B**) hsa-mir-1-1 and SNRPD1. (**C**) hsa-mir-200b and *CFL2*. The analysis was performed by Pearson’s correlation, where inverse correlations were considered significant at *p* ≤ 0.05, R ≤ − 0.3. The blue line represents the regression line, and the gray shading represents the 95% confidence interval. IGC = intestinal gastric cancer; DEMs = differentially expressed microRNAs; DEGs = differentially expressed genes.

We performed correlation analyses to verify and strengthen the evidence of these interactions. In IRC, there was no significant inverse correlation between hsa-mir-26b and *SNRPD1* (R = −0.037, *p* = 0.62) (Figure 11A), but there was a significant inverse correlation between hsa-mir-1-1 and *SNRPD1* (R = −0.33, *p* < 0.001) (Figure 11B). For RRC1, we observed a strong inverse correlation between hsa-mir-200b and *CFL2* (R = −0.66, *p* < 0.001), strengthening the evidence of this regulatory interaction (Figure 11C). Importantly, the upregulated DEM hsa-mir-200b hub and its downregulated target DEG/DEP *CFL2* hub in RRC1 were related not only to pathological T stage but also to OS. Overall, these results indicate that this regulatory interaction may be an important component in the pathogenesis of IGC.

## 4. Discussion

Altered expression of noncoding RNAs, genes, and proteins are well-known players driving tumorigenesis. These molecules do not function individually; rather, they act through network complexes, connecting important regulatory biological processes. In GC, few studies have used integrated biological data, and the main focus has been on noncoding RNA-mRNA interactions [11,12,13,14], neglecting the idiosyncrasies of each GC subtype, which is an important bottleneck in GC research as the subtypes are molecularly different entities, and excluding proteomic data, which are the real result of gene expression. Nevertheless, identifying how those molecules are related is an important step in identifying key components in the development of IGC. Thus, the main purpose of this study was to integrate data obtained from DEMs, DEGs, and DEPs and explore their regulatory relationships in a specific GC subtype, the IGC, which, as far as we know, has not been done to date. Accordingly, we performed comparative analysis between patient samples from IGC tumors and adjacent nontumor tissues using different omics approaches (miRNomics, transcriptomics, and proteomics). The results were subjected to extensive bioinformatics analysis carried out to integrate data from the expression profiles of DEMs, DEGs, and DEPs and extract relevant biological information in the context of the disease to obtain a comprehensive overview of the main components involved in gene regulation of the IGC subtype.

The data integration revealed three interaction networks regarded as regulatory circuits: RRCI and RRC2 characterized by upregulated DEMs leading to downregulated DEPs and IRC characterized by downregulated DEMs leading to upregulated DEPs. These circuits enabled us to identify important biological alterations and key regulators in IGC development. Regulatory circuits were constructed based on overlapping identification of DEGs and DEMs, in which information on validated regulatory DEMs was included based on the classical regulatory mechanism of microRNAs. Comprehensive enrichment analysis of the network components was performed, and in the three regulatory circuits, they were related to similar pathways associated with relevant hallmarks of cancer, such as the cell cycle, cell death, and the immune response, indicating their importance in IGC carcinogenesis. Interestingly, RRC1 and RRC2 shared 73% of the set of microRNAs, including upregulation of oncomir families, such as miR-17, miR-19, and mir-25 [35,36,37]. Furthermore, we observed downregulation of known families of tumor-suppressor miRNAs in IRC, such as the miR-26, miR-29, mir-30, and let-7 families [36]. Conversely, the hubs, including DEGs and DEPs, were very specific for each circuit. This indicates that different mechanisms of gene regulation impact similar biological functions.

Hub analysis was used to identify key regulators in the network, which revealed 15 DEG/DEP hubs (five for each regulatory circuit) and 27 DEM hubs (10 in RRC1, 12 in RRC2, and 5 in IRC).

Overall, identifying and characterizing hubs is a very important step in understanding important components in biological networks. It is known that deleting highly connected hubs has a high probability of lethality to organisms (centrality–lethality rule); this is due to its functional importance connecting several nodes related to multiple pathways in biological systems that makes them essential [38]. This information is central not only to understanding the network structure but also to predicting the magnitude of changes that can occur if a given hub is subjected to management as they are important for network maintenance [39]. This context needs to be accounted for during the discovery of potential targets for patient treatment. Finding hubs that are manageable and with alterations that are specifically related to tissues or cells involved in the disease has the potential to increase the efficiency of treatment. Therefore, during our study, we focused on the relationship between hubs and relevant aspects of IGC patient clinicopathology to highlight important hubs in IGC pathogenesis.

We observed that some of our hubs had high diagnostic potential. A total of 11 DEM and 11 DEG hubs showed good diagnostic test power (AUC > 0.7). The hub with high diagnostic potential was downregulated hsa-miR-195-5p (AUCs = 0.9409) in IRC. hsa-miR-195-5p downregulation has been found to be a potential biomarker for lung and breast cancer [40,41]; this DEM also has good potential as a biomarker in GC. In accordance with our results, hsa-miR-195-5p expression was significantly downregulated in the tissues and serum of GC patients [42]. hsa-miR-195-5p is related to regulation of the Wnt/β-catenin pathway [42], the epithelial-to-mesenchymal transition [43], and multidrug resistance [44,45]. However, expression of hsa-miR-195-5p related to different subtypes of GC requires additional study.

Further investigation showed that seven hubs were also related to the T stage in IGC. We observed that expression of the upregulated hub hsa-miR-15b of RRC1 decreased with increasing T stage. In GC, overexpression of hsa-miR-15b-5p has been verified to promote carcinogenesis, and a high plasma level of miR-15b-5p correlates with distant tumor metastasis [46]. Additionally, in accordance with our results, hsa-miR-15b-5p was found to be upregulated as a key regulator in a study involving 1000 GC samples. However, it could not predict the stage of GC, which may be related to the fact that the authors did not consider subtypes separately [47]. Our results indicate that expression of this DEM in IGC is required at different levels during tumor development. Additionally, this hub might serve as an early prognostic marker due to its relatively high expression in early T stages, which may be related to tumor establishment.

On the other hand, expression of the upregulated hsa-miR-let-7d of RRC2 increased with increasing T stage. miRNAs of the let-7 family are known for their tumor-suppressor functions and are commonly repressed in tumors [48]. They inhibit tumor growth and metastasis [49,50,51]. However, in accordance with our results, Gao and colleagues [52] observed that expression of hsa-miR-let-7d-5p was upregulated in CG cell lines, promoting carcinogenesis by targeting and decreasing expression of PR/SET domain 5 protein (PRDM5), a protein that modulates important processes, such as growth, differentiation, and apoptosis [53], which was also downregulated in our data (Supplementary Table S2), though not identified as a hub. These results indicate that in IGC, hsa-miR-let-7d has uncommon behavior and may act as a therapeutic and clinical biomarker.

We also noted that components in the regulatory circuits seem to work together, contributing to clinical outcomes. Interestingly, in IRC, the downregulated DEM hubs hsa-mir-26b and hsa-mir-1-1 potentially failed to inhibit expression of the potential target *SNRPD1,* which was upregulated at both the mRNA and protein levels. Moreover, we observed that with increasing T stage, only expression of hsa-mir-26b decreased, whereas that of *SNRPD1* increased. Corroborating our data, high expression of small nuclear ribonucleoprotein Sm D1 (*SNRPD1*) was found at both the gene [54] and protein levels (in serum) [55] in GC. In turn, significant downregulation of hsa-mir-26b-5p has been observed in *Helicobacter* pylori-infected GC cells and tissues, promoting proliferation in vitro and in vivo, and is related to poor outcome in GC patients [56].

The results that caught our attention were the potential regulatory control of downregulated *CFL2* by upregulated hsa-miR-200b in RRC1. These hubs showed good diagnostic value (AUC = 0.7411 and 0.8088 for hsa-miR-200b and CFL2, respectively), with the most significant difference in expression through T1 × T4 stages (0.00011 and 0.00017 for hsa-miR-200b and CFL2, respectively) and a significant impact on the OS of IGC patients. Finally, their expression exhibited a strong inverse correlation (R = −0.66, *p* < 0.001), indicating a possible important regulatory pair in IGC carcinogenesis.

The hsa-miR-200b hub is a component of the conserved miR-8/miR-200 family [57], which was enriched in RRC1. Interestingly, all components of this family, except miR-429, were upregulated DEMs in our study (hsa-miR-200a, hsa-miR-200c, and hsa-miR-141). The miR-200 family plays a major role in EMT and metastasis suppression by repressing ZEB1 (downregulated DEG in our study—Supplementary Table S2) and ZEB2, transcription factors that promote tumor invasion and metastasis by inducing EMT through E-cadherin repression [58,59]. Our results showed that although the hsa-miR-200b hub was upregulated in IGC, its expression decreased with increasing T stage. Considering the importance of hsa-mir-200b in EMT, this may be a crucial event in promoting tumorigenesis in IGC.

The miR-200 family is upregulated in some cancers, such as bladder cancer, melanoma, and ovarian cancer, though its downregulation is observed in others, including renal cell carcinoma and hepatocellular carcinoma [60,61,62]. In Asian populations, expression of hsa-miR-200c and mainly hsa-miR-200b is reported to be downregulated in GC tissues and related to poor OS [63,64,65,66]. However, hsa-miR-200b and hsa-miR-200c are also upregulated in brain metastases in GC patients [67] and serum samples, where high levels of hsa-miR-200c are also related to short OS [68,69]. In our study, high expression of hsa-miR-200b was observed in IGC patients and correlated with OS as a protective factor. These apparently contradictory observations warrant further investigation as other factors may contribute to these disparities, such as sample size, cancer subtype, and detection method. Moreover, risk factors such as *H. pylori* infection, drinking, smoking, and the molecular background of each population impact the development and progression of GC. Altogether, these data suggest that regulation of tumor initiation and progression by the miR-200 family in IGC may be related to factors other than expression alone.

In our work, *CFL2* was one of the top hubs, being the target of several DEMs, including DEM hubs, mainly hsa-miR-200b, and its expression showed a strong inverse correlation, indicating that this microRNA may be a major regulator of *CFL2* in IGC patients. The impact of high *CFL2* on survival and its association with advanced T stage render this hub an important player in IGC carcinogenesis.

Cofilin 2 (*CFL2*) is a member of the actin-depolymerizing factor (ADF)/cofilin family [70]. It is related to regulation of cytoskeletal dynamics through its function as an actin-binding and regulatory protein, playing a central role in catalyzing actin polymerization and depolymerization through its actin-severing activity [71]. *CFL2* also displays important functions in muscle regeneration and maintenance [70,71]. Mutations in *CFL2* cause myopathies in humans, and its deletion is associated with lethality in mice due to high muscle deficiency and actin accumulation within myofibrils [72,73,74].

Data on *CFL2* in malignancies are limited. However, our results indicate that compared to adjacent nontumor samples, CFL2 is downregulated at both the mRNA and protein levels in IGC patient tumors, similar to what was observed in pancreatic cancer [75]. In breast cancer, upregulation of *CFL2* was detected in aggressive cell lines and tissues [76]; similar findings were also observed in prostate cancer, promoting proliferation and migration in cell lines [77]. This information is evidence that *CFL2* expression is context dependent.

In GC, there are few studies related to *CFL2* expression; overall, *CFL2* has not been the focus of research to date and rather a secondary finding. Nonetheless, supporting the findings in our network, a constructed network of differentially expressed lncRNA–miRNA–mRNA in GC patients identified *CFL2* as one of the downregulated genes targeted by hsa-miR-20a-5p (upregulated hub in RRC1 also targeting *CFL2*—Supplementary Table S3) and hsa-miR-148a-3p (downregulated DEM in our work—Supplementary Table S3) [78]. Data from gastric cancer cells SGC7901 and BGC823 transfected with miRNA-194 (upregulated in our study targeting *CFL2*) identified downregulated *CFL2* as one of the top 23 coding genes most downregulated by miR-194 mimics in GC [79]. Furthermore, other studies reinforce that *CFL2* regulation seems to be highly related to miRNA activity: *CFL2* was identified as a validated target of miR-16 [80] and miR-375 [81]. In addition to *CFL2* not being the main focus in these works, these studies have limitations as the authors did not discriminate between samples regarding histological subtype, which hinders interpretation of the results or comparison with our data. Regardless, as we observed, *CFL2* expression is intricately regulated and seems to be related to epigenetic control through noncoding RNA mechanisms.

Interestingly, through survival analysis, we found that patients who showed greater downregulation of *CFL2* had better OS than the other group. This result is supported by the findings in GC cell lines by Bian and colleagues [82], who verified that *CFL2* knockdown inhibits proliferation and migration of MGC803 cells and that high *CFL2* expression is associated with poor prognosis in patients with GC [82], demonstrating the oncogenic function of *CFL2*. According to our data, expression of *CFL2* in patients with advanced T stage was higher than that in patients with early stages. The same result was observed in breast cancer tumor tissue, where CFL2 expression increased with tumor grade [76]. Hence, regulation of *CFL2* may be required at some level for IGC carcinogenesis.

Overall, the findings suggest that dynamic expression of *CFL2* in IGC patients may be necessary for tumor progression and that this control may be performed by decreased expression of its repressor hsa-miR-200b. How *CFL2* expression dynamics are involved in promoting tumor progression must be clarified by further studies. However, our study suggests that regulation of *CFL2* by hsa-miR-200b is a dynamic process during tumor progression and that this control plays essential roles in IGC development.

## 5. Conclusions

Our results showed, for the first time, the impact of the use of integrative analysis of microRNAs, mRNAs, and proteins applied to IGC investigation. By using high-throughput approaches and bioinformatics-based tools for biological characterization, our study identified important DEMs, DEGs, and DEPs involved in three complex regulatory circuits that participate in important biological pathways, such as the cell cycle, cell death, and the immune response, potentially regulated by regulatory circuits through different mechanisms. Additionally, the hubs are associated with relevant aspects of IGC patient clinicopathology, with good potential as diagnostic, prognostic, and tumor development biomarkers. Furthermore, we noted that components in the regulatory circuits appear to operate together to contribute to clinical outcomes, such as the potential regulatory control of downregulated *CFL2* by upregulated hsa-miR-200b, which is a likely target of future investigations. In summary, our study provides a comprehensive investigation of IGC gene regulation, and the identified hubs are good targets for future investigation to understand disease development and progression as well as to search for pharmacological targets.

## Figures and Tables

**Table 1:** Clinicopathological data of IGC patients from the TCGA dataset.

Clinicopathological Features		Cohort
State	Primary tumor	180
	Normal tissue	18
Gender	Male	119
	Female	61
Ethnicity	White	100
	Asian	51
	Black or African American	4
	Not reported	25
Major pathologic TNM stages	Stage I	31
	Stage II	62
	Stage III	62
	Stage IV	12
Pathologic T stage	T1	11
	T2	50
	T3	69
	T4	44
Pathologic N stage	N0	65
	N1	43
	N2	33
	N3	29
Pathologic M stage	M0	166
	M1	6
Molecular subtype	Chromosomal instability	108
	Microsatellite instability	44
	Genomically stable	14
	Epstein–Barr virus (EBV)+	14

**Table 2:** Top DEG/DEP hubs in each regulatory circuit.

Hub	Score (DEGs/DEPs)	Number of Regulators	Regulatory Circuit	Expression in IGC DEG/DEP
CDKN1A	102.0	102	RRC1	Down/Down
PPP1CB	29.0	29	RRC1	Down/Down
CKB	27.0	27	RRC1	Down/Down
TTC9	18.0	18	RRC1	Down/Down
CFL2	47.0	47	RRC1	Down/Down
PCNA	25.0	25	RRC2	Up/Down
QSOX2	17.0	17	RRC2	Up/Down
APOH	14.0	14	RRC2	Up/Down
SPAG5	12.0	12	RRC2	Up/Down
SEPTIN14	12.0	12	RRC2	Up/Down
SNRPD1	19.0	19	IRC	Up/Up
BID	12.0	12	IRC	Up/Up
UBE2C	11.0	11	IRC	Up/Up
HSPE1	10.0	10	IRC	Up/Up
OLFM4	5.0	5	IRC	Up/Up

**Table 3:** Top DEM hubs in each regulatory circuit.

Hub	Number of Targets	% of DEGs/DEPs Targeted in the Circuit	Regulatory Circuit	DEM Expression in IGC
hsa-miR-210-3p	14	30.4	RRC1	Up
hsa-miR-200b-3p	9	19.6	RRC1	Up
hsa-miR-335-5p	7	15.2	RRC1	Up
hsa-miR-20a-5p	7	15.2	RRC1	Up
hsa-miR-148b-3p	7	15.2	RRC1	Up
hsa-miR-17-5p	6	13.0	RRC1	Up
hsa-miR-15b-5p	6	13.0	RRC1	Up
hsa-miR-15a-5p	6	13.0	RRC1	Up
hsa-miR-106b-5p	6	13.0	RRC1	Up
hsa-miR-92a-3p	6	13.0	RRC1	Up
hsa-miR-17-3p	3	37.5	RRC2	Up
hsa-miR-522-5p	3	37.5	RRC2	Up
hsa-miR-92a-3p	3	37.5	RRC2	Up
hsa-let-7d-5p	2	25.0	RRC2	Up
hsa-miR-18a-5p	2	25.0	RRC2	Up
hsa-miR-3653-5p	2	25.0	RRC2	Up
hsa-miR-373-3p	2	25.0	RRC2	Up
hsa-miR-4745-5p	2	25.0	RRC2	Up
hsa-miR-20a-5p	2	25.0	RRC2	Up
hsa-miR-106b-5p	2	25.0	RRC2	Up
hsa-miR-1304-5p	2	25.0	RRC2	Up
hsa-miR-502-3p	2	25.0	RRC2	Up
hsa-miR-23b-3p	4	33.3	IRC	Down
hsa-miR-1-3p	4	33.3	IRC	Down
hsa-let-7b-5p	3	25.0	IRC	Down
hsa-miR-195-5p	3	25.0	IRC	Down
hsa-miR-26b-5p	3	25.0	IRC	Down

## Data Availability

IGC microRNA and mRNA data are available at https://portal.gdc.cancer.gov under TCGA-STAD accessed on 5 March 2023. Proteomic data can be found at https://content.iospress.com/articles/cancer-biomarkers/cbm203225.

## Supplementary Materials

The following supporting information can be downloaded at: https://www.mdpi.com/article/10.3390/cancers15225374/s1, Supplementary Table S1: Clinicopathological data of patients in the proteomic study. Supplementary Table S2: Complete list of selected DEGs identified in IGC TCGA patients. Supplementary Table S3: Complete list of selected DEMs identified in IGC TCGA patients.
